# GSH Protects the *Escherichia coli* Cells from High Concentrations of Thymoquinone

**DOI:** 10.3390/molecules27082546

**Published:** 2022-04-14

**Authors:** Robert Łyżeń, Grzegorz Gawron, Leszek Kadziński, Bogdan Banecki

**Affiliations:** Intercollegiate Faculty of Biotechnology UG and MUG, University of Gdansk, Abrahama Str. 58, 80-307 Gdansk, Poland; robert.lyzen@ug.edu.pl (R.Ł.); grzegorz.gawron@phdstud.ug.edu.pl (G.G.); bogdan.banecki@ug.edu.pl (B.B.)

**Keywords:** *Escherichia coli*, thymoquinone, glutathione, ROS, HPLC

## Abstract

The aim of the present study was to evaluate the potential protective effect of glutathione (GSH) on *Escherichia coli* cells grown in a high concentration of thymoquinone (TQ). This quinone, as the main active compound of *Nigella sativa* seed oil, exhibits a wide range of biological activities. At low concentrations, it acts as an antioxidant, and at high concentrations, an antimicrobial agent. Therefore, any interactions between thymoquinone and glutathione are crucial for cellular defense against oxidative stress. In this study, we found that GSH can conjugate with thymoquinone and its derivatives in vitro, and only fivefold excess of GSH was sufficient to completely deplete TQ and its derivatives. We also carried out studies on cultures of GSH-deficient *Escherichia coli* strains grown on a minimal medium in the presence of different concentrations of TQ. The strains harboring mutations in gene *ΔgshA* and *ΔgshB* were about two- and fourfold more sensitive (256 and 128 µg/mL, respectively) than the wild type. It was also revealed that TQ concentration has an influence on reactive oxygen species (ROS) production in *E. coli* strains—at the same thymoquinone concentration, the level of ROS was higher in GSH-deficient *E. coli* strains than in wild type.

## 1. Introduction

Aerobic conditions and respiration, cellular metabolism, host–pathogen interactions, or antibiotic treatment expose bacteria to a variety of redox-active species, such as reactive oxygen, electrophile, nitrogen, chlorine, and sulfur species [1]. Their high concentration may affect the cellular redox balance and damage cellular macromolecules such as proteins, nucleic acids, lipids, and carbohydrates. Therefore, living cells have developed many strategies to defend against oxidative stress. One of these strategies is the use of low-molecular-weight (LMW) thiols. The LMW thiols are important intracellular reductants and antioxidants, helping to maintain essential –SH groups of enzymes in their reduced state. In many Gram-negative bacteria such as *Escherichia coli* and eukaryotes, the active LMW thiol is glutathione (GSH) [2]. GSH is synthesized from L-glutamate, L-cysteine, and glycine in two consecutive steps, catalyzed by γ-glutamyl-cysteine synthase, and glutathione synthase [3]. Under physiological conditions, GSH can reduce different compounds, such as lyophilic molecules, which can freely cross the plasma membrane [4,5,6,7]. In *E. coli*, glutathione is synthesized by two synthases encoded by *gshA* and *gshB* genes. The deletion of *gshA* or both *gsh* genes does not affect the growth rate in the Luria-Bertani (LB) medium, but the deletion of *gshB* alone reduced it by half. However, grown in LB broth, they exhibit a presence of GSH in the cells [8].

Thymoquinone (2-isopropyl-5-methylbenzo-1, 4-quinone) is one of the active compounds of oils from seeds of *Nigella sativa* [9]. Oils and TQ exhibit a wide range of pharmacological activities including antioxidant, anticancer, anti-inflammatory and immunomodulatory, antimicrobial, neuroprotective, cardioprotective, gastroprotective, hepatoprotective, antidiabetic, etc. [10,11]. In the cell, TQ can be a potent redox-active compound that can undergo enzymatic and nonenzymatic redox cycling with its corresponding semiquinone radical; as a result, superoxide anion radicals can be generated. The accumulation of them might contribute to the prooxidant effect of TQ [12,13,14]. TQ also reacts chemically with GSH: this reaction is very fast and occurs under physiologically stable conditions. A 10-fold excess of GSH was required to completely deplete TQ [4]. The end products are glutathionyl–dihydrothymoquinone (GS-DHTQ) and small amounts of dihydrothymoquinone, reduced substances that can act as scavengers for hydroxyl and carbon-centered radicals. Importantly, both are more powerful free radical scavengers than TQ [4,13,15]. On the other hand, quinone thiol ethers such as GS-DHTQ can be reduced by reductase to autooxidizable semiquinone–GSH conjugates, greatly enhancing H_2_O_2_ formation [12].

This study was undertaken to test the protective role of GSH as a potential therapeutic molecule against the antimicrobial activity of thymoquinone [10,11]. TQ is a quinone and occurs naturally in plants such as *N. sativa*, *T. vulgaris*, etc., constituting a diet component. Recent data indicate that, unlike the others, the antimicrobial activity of thymoquinone is most likely related to the production of reactive oxygen species that can damage DNA and other cell structures [16,17]. This hypothesis was also confirmed by the results of our studies. We found that in growing cultures of *E. coli* strains supplemented with TQ at a concentration close to or above the minimum inhibitory concentration (MIC), the levels of ROS increased dramatically. However, the toxic effect of TQ on GSH-deficient *E. coli* strains was at least two times stronger than that of wild type, suggesting that GSH may play an important role in the protection of bacterial cells against TQ. Moreover, this protection seems to be enhanced, as GSH can also conjugate with TQ derivatives and potentially neutralize them.

## 2. Results

### 2.1. Interaction of TQ with Organosulfur Compounds

**Figure 1:**
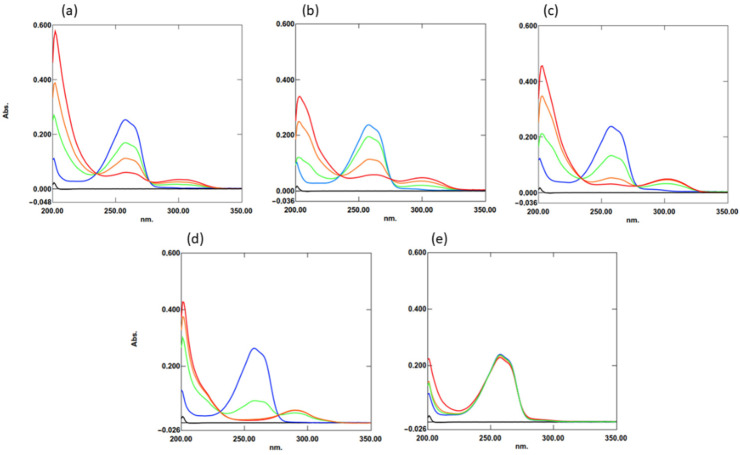
Analysis of UVVIS spectra of thymoquinone interaction and increasing concentrations of (**a**) glutathione, (**b**) cysteine, (**c**) N-acetylcysteine, (**d**) DTT, and (**e**) cystine. The organosulfur compounds concentration were at 0 µM (blue line), 25 µM (green line), 50 µM (orange line), 100 µM (red line), and reaction buffer as a blank (black line). The analyses were performed in triple independent repetitions.

Khalife and Lupidi [4] showed that TQ can react quickly and spontaneously with GSH via –SH moiety. They suggested that this was due to the addition of a sulfur atom to the quinone ring generating a quinone thiol ether. Therefore, to answer the question of whether this is a general rule for organosulfur compounds, GSH and four other compounds were tested. These compounds were cysteine, N-acetylcysteine, dithiothreitol (DTT), and cystine (which contains disulfide bonds) as a control. For this procedure, increasing concentrations of the compounds were added to 20 µM TQ in phosphate buffer. Then, the mixtures were incubated at 25 °C for 5 min, and spectra in the range of 200–400 nm were collected. Cysteine, N-acetylcysteine, and DTT (which contains a thiol group) reacted quickly, and TQ depletion was directly proportional to their concentrations (Figure 1). On the other hand, cystine did not react.

**Figure 2:**
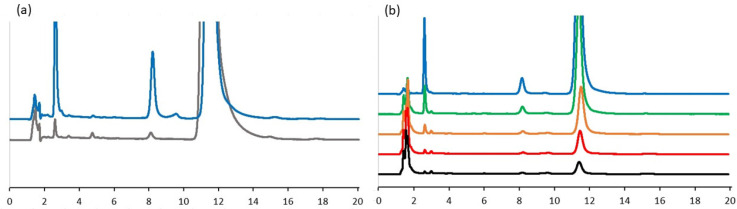
The level of thymoquinone degradation in minimal medium after 24 h incubation at room temperature (**a**) and the rate of TQ and its derivatives depletion depending on increasing GSH concentrations (up to 4-fold excess) (**b**). Description of the line color: TQ (grey line); TQ after 24 h of natural degradation (blue line); TQ mixed with GSH in the ratio: 1:1 (green line); 1:2 (orange line), 1:3 (red line); 1:4 (black line). The analyses were performed in triple independent repetitions.

As TQ in water solution is easily degraded under oxidizing conditions and light [18], the TQ solution was prepared in a minimal medium and left for 24 h at room temperature (~22 °C). In the first step, the level of TQ degradation was analyzed by using HPLC chromatography. The analysis showed that TQ degraded to several derivatives (two dominated), which could be separated on C-18 resin (Figure 2a). Next, increasing concentrations of GSH were added to this mixture, and after short incubation at room temperature, the mixtures were analyzed. Regardless of the type of compounds, they were all bound by GSH (Figure 2b), and about a fivefold excess of GSH was sufficient to completely deplete the TQ and its derivatives.

### 2.2. Sensitivity of GSH-Deficient Strains to TQ

**Figure 3:**
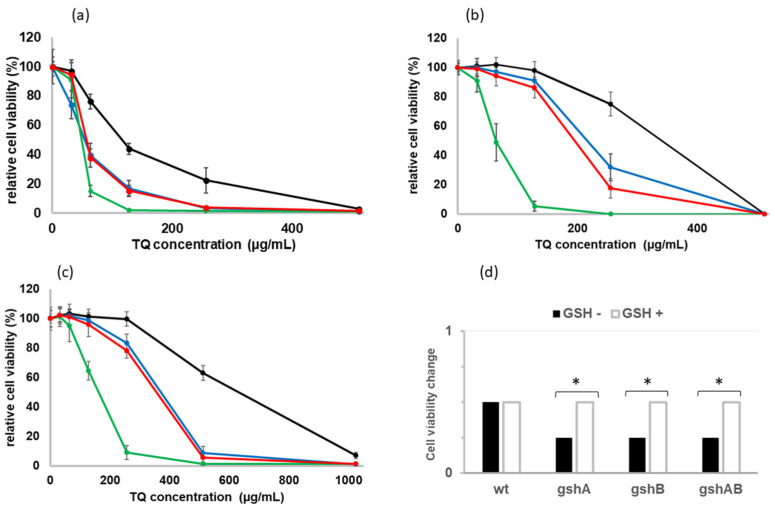
The effect of TQ concentration on *E. coli* cells viability of wild type (black line), Δ*gshA* (blue line), Δ*gshB* (green line), and Δ*gshA*Δ*gshB* (red line) in minimal medium (**a**), minimal medium with the addition of 5 mM GSH (**b**), LB medium (**c**), and fold change in cell viability; cell viability in LB was used as a control (**d**). Mean values with deviation for at least three replicates were plotted. Asterisk mark—statistically significant differences (*p* < 0.05 in *t*-test), compared with control.

In the light of the fact that GSH reacts at a similar rate as that of TQ and its derivatives, it is highly probable that it may better protect *E. coli* cells from their high concentrations and counteract the lethal effect of TQ. As the *E. coli* may import GSH into the cell [6], studies have been performed on two types of broth: minimal medium and rich medium (LB) which contains GSH. First, the studies on cultures of GSH-deficient *E. coli* strains grown in a minimal medium in the presence of different concentrations of TQ (0–512 µg/mL) were carried out. After overnight incubation at 37 °C with shaking, the number of living cells was analyzed by spectroscopy (Figure 3a), and the number of colonies formed on agar plates was counted. TQ concentrations where at least 99.0% of the cells were dead were taken as MICs. The strains harboring mutations in genes Δ*gshA* and Δ*gshB*, were about two- and fourfold more sensitive, 256 and 128 µg/mL, respectively, than the wild type. Interestingly, the Δ*gshA*Δ*gshB* double mutant was only about twofold more sensitive than the wild type. Since *E. coli* can import GSH from the medium into the periplasm [12] and at least partially compensate for its lack in the cell, all strains were grown to the exponential phase and supplemented with 5 mM GSH. Then, to reduce the impact of GSH present in the medium, the cultures were centrifuged and resuspended in a fresh MM medium. The accumulated GSH in the cells significantly increased the cell viability of all strains, especially those carrying mutations in the *gsh* genes (Figure 3b), which were about 2 times less sensitive to TQ than in the minimal medium (Figure 3d; *p* < 0.05). Similar effects were observed in the LB medium (Figure 3c).

### 2.3. Formation of ROS in the Presence of TQ in the Culture

**Figure 4:**
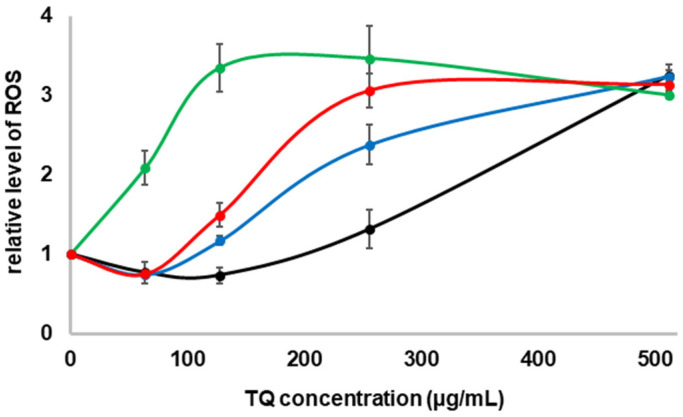
The effect of TQ concentration on ROS production in *E. coli* strains cultures: wild type (black line), Δ*gshA* (blue line), Δ*gshB* (green line), and Δ*gshA*Δ*gshB* (red line). Mean values with deviation for at least three replicates were plotted.

At higher concentrations, TQ may act as a prooxidant, inducing ROS synthesis and damaging or even killing bacterial cells [19,20]. This fact and the different sensitivity of GSH-deficient *E. coli* strains suggested that in the growing cultures of strains Δ*gshA* and/or Δ*gshB* in the presence of TQ, ROS could be formed earlier at doses that were lethal to them. To test this possibility, after 3 h incubation at 37 °C with shaking, DCFH–DA was added to the growing cultures supplemented with increasing concentrations of TQ, and the incubation was continued in the dark for 1 h. Next, the level of ROS was measured by spectrofluorimetry. At the lower TQ concentration, 64–128 µg/mL for wild type and 64 µg/mL for Δ*gshA* and Δ*gshA*Δ*gshB*, the ROS level was even lower than that in the culture without TQ addition (Figure 4). At a concentration equal to half the MIC in all cultures, the ROS level began to increase and peaked at the MIC concentration, except for Δ*gshA* for which the concentration was slightly higher.

## 3. Discussion

Host–pathogen interactions, aerobic respiration, cellular metabolism, and antibiotic treatment expose bacteria to a variety of redox-active species [1]. These factors affect the cellular redox balance and lead to damage to cellular macromolecules such as proteins, nucleic acids, lipids, and carbohydrates. Therefore, living organisms have developed many defense mechanisms against them. One of them is the use of glutathione (GSH), which belongs to the low-molecular-weight (LMW) thiols. GSH is essential to maintaining an appropriate thiol/disulfide redox potential or activity of thiol-dependent enzymes [2]. Under physiological conditions, the concentration of GSH is from 10- to 100-fold higher than that in oxidized form, and therefore, it may be able to reduce different compounds, such as lyophilic molecules, which can freely cross the plasma membrane due to their chemical nature [4,6]. In addition, in aerobically growing *E. coli* cells, more than 10% of the total GSH is excreted into the medium, where its concentration is maintained at an approximately constant level during culture growth [6]. The GSH-dependent detoxification pathway involves enzymatic components and produces mercapturic acid; a final product is usually a nontoxic N-acetylated cysteine–toxin conjugate that is excreted from the cell [21].

It has been shown that intracellular glutathione is involved in several reactions associated with the metabolism of quinones such as menadione [22]. It can directly form a conjugate with menadione and excrete it from the cell into the medium. GSH can also react readily with thymoquinone and reduce it nonenzymatically to glutathionylated dihydrothymoquinone [4]. A similar mechanism is also observed with fluoroquinolone antibiotics such as ciprofloxacin [23]: In *E. coli* cells, exogenous GSH reversed the negative effects of ciprofloxacin by neutralizing ciprofloxacin-induced oxidative stress and increasing its efflux. Furthermore, as we showed, GSH can also conjugate with TQ derivatives (Figure 2), and the rate of this process seems to be similar to that of TQ. About a fivefold excess of GSH was sufficient to completely deplete the TQ and its derivatives (Figure 2b). Similar results were obtained by Khalife and Lupidi [4]. Additionally, because TQ can also bind to other thiol-containing compounds (Figure 1), protecting cells from its effects should be even more effective.

For cultures in MM medium supplemented with TQ below the half of MIC value for each strain (Figure 4), the ROS level was even lower than the levels in the controls growing without TQ. This is consistent with previous data. It was shown that the reduced forms of TQ in lower concentrations have great antioxidant properties and are good scavengers of hydroxyl and carbon radicals [15,24]. Further, the end product of TQ and GSH conjugation, GS-DHTQ, is a more powerful free radical scavenger than TQ [4,13]. On the other hand, at higher concentrations, TQ may act as a pro-oxidant [19,20], inducing the synthesis of ROS [16,17]. Similar effects were seen in this study. In growing cultures, when TQ concentration exceeded half of the MIC value (Figure 3a), 512 µg/mL for wild type, 256 µg/mL for *ΔgshA* and double mutant, and 128 µg/mL for *ΔgshB*, the ROS level quickly increased. For all strains, the ROS level was about threefold higher at the MIC values (Figure 4). That number of free radicals was sufficient to kill each of the strains. The mechanism of TQ action is likely similar to that of menadione. As was shown previously, increased menadione toxicity resulted in the depletion of intracellular GSH [22], which increased the formation of semiquinone radicals. The accumulation of superoxide radicals might contribute to the prooxidant effects of menadione [25] and TQ [4]. Quinone thiol ethers such as GS-DHTQ can be reduced by reductase to autooxidizable semiquinone-GSH conjugates and greatly enhance H_2_O_2_ formation [12]. However, this does not explain the discrepancy in reducing cell density at lower TQ concentrations (Figure 3) when ROS levels were also low (Figure 4). These differences may be due to a different mechanism described by Ahmad et al. [26]. They showed that lower concentrations of TQ (<150 µM) strongly reduced the growth of *E. coli* cells by inhibiting the activity of membrane ATPase but did not kill them.

The *E. coli* strains grown on the minimal medium were more sensitive to TQ than those grown on the LB medium (Figure 3). This is because they can import GSH, which is a component of the LB medium. *E. coli* cells contain several glutathione transporters encoded by the genes *yliA*, *yliB*, *yliC*, and *yliD* [27], and in cells grown in LB broth, the presence of GSH in the cytoplasm has been observed [8]. This was also confirmed by studies in which GSH-deficient *E. coli* strains were grown on a minimal medium supplemented with GSH (Figure 3b). Furthermore, Helbig et al. [8] showed that the deletion of *gshA* or *gshA/gshB* genes did not affect the growth rate in the LB medium, but the deletion of *gshB* alone reduced it by half. Similar correlations were observed for both broths used. This may explain the increased sensitivity to TQ of the Δ*gshB* strain compared with Δ*gshA* and double mutant (Figure 3 and Figure 4).

## 4. Materials and Methods

### 4.1. Chemicals

Chemicals: thymoquinone (TQ), cysteine, cystine, N-acetylcysteine, dithiothreitol (DTT), reduced glutathione (GSH) were obtained from Merck (Darmstadt, Germany).

### 4.2. Bacterial Strains

*Escherichia coli* W3110 strain (F- λ- *rph-1* INV(*rrnD*, *rrnE*)) and its three derivatives: ECA390 (Δ*gshA*; deletion in gene encoding: the γ-Glu-Cys synthetase); ECA391 (Δ*gshB*; deletion in gene encoding: the GSH synthetase) and double mutant ECA460 (Δ*gshA* Δ*gshB*) were a gift from prof. Nies (Germany) [8].

### 4.3. The Reaction of TQ with Thiol Group

The analysis of the interaction of TQ with a thiol group was carried out in in vitro conditions. A set of five organosulfur compounds including cysteine, N-acetylcysteine, DTT, GSH (all containing thiol group), and cystine (which contains disulfide bonds) were selected and dissolved to the final concentration of 400 µM. The reaction of TQ was performed at 25 °C in phosphate buffer pH 7.0, containing 20 µM TQ and the organosulfur compounds added to final concentrations of 0, 10, 20, and 40 µM. After 5 min of incubation, the spectra (in the range of 200–400 nm) were recorded and processed using a PerkinElmer spectrophotometer (PerkinElmer, Waltham, MA, USA).

### 4.4. Minimum Inhibitory Concentration Determination

To determine the minimum inhibitory concentration (MIC) of TQ (0 to 512 μg/mL), the method described by Gawron et al. [28], with some modifications, was used. Briefly, an overnight culture (37 °C) of the tested strains was diluted 10-fold in fresh minimal medium, with and without the addition of 5 mM GSH (MM medium, according to [29]), and Luria–Bertani (LB) medium and then incubated (37 °C) until they reached the exponential growth phase. To minimize the effect of GSH present in the minimal medium, cells cultured with GSH were centrifuged and resuspended in the same volume of fresh MM medium. Serial twofold dilutions of TQ in MM medium or LB were prepared in a 15 mL plastic tube (1.9 mL). The inocula (100 μL) containing 10^6^ cfu/mL of each reference strain were added. The sterility control of the medium (no inoculum added) was carried out. After incubation for 24 h at 37 °C, bacterial growth was evaluated. First, 100 μL of cultures were spread on agar plates (incubation 24 h at 37 °C), and then the cell density was estimated by using a PerkinElmer Lambda 25 spectrophotometer (PerkinElmer, Waltham, MA, USA). The MIC was defined as the concentration that completely inhibited visible cell growth during a 24 h incubation period at 37 °C.

### 4.5. Analysis of Interaction of GSH with TQ Derivatives by HPLC Chromatography

Chromatographic analyses were performed using a PerkinElmer series 200 HPLC system (PerkinElmer, Waltham, MA, USA) comprising a quaternary LC pump, autosampler, column oven, and a UV detector. Reaction conditions, sample preparation, and analysis were performed as described by Khalife and Lupidi [4], with small modifications. Briefly, GSH was dissolved in PBS buffer and mixed with solutions of degraded TQ. After 2 min of incubation at room temperature (~22 °C), the samples were mixed with methanol (1:1, *v*/*v*) and filtered through a 0.45 mm Millipore filter. The volume injected was 20 μL. The isocratic elution on an Agilent Eclipse XDB-C18 column (3.5 µm, 150 × 4.6 mm) (Agilent Technologies, Santa Clara, CA, USA) was performed at a flow rate of 1.0 mL/min, with the mobile phase composed of water:methanol:2-propanol (50:45:5 *v*/*v*). Analyses were performed at room temperature. UV monitoring of the eluted solutions was carried out at 254 nm.

### 4.6. ROS Analysis in the Cultures Treated by TQ

Intracellular amounts of ROS were analyzed by fluorescence spectroscopy after being reacted with 2′,7′-dichlorodihydrofluorescein diacetate (DCFH-DA; Merck, Germany) according to the method described by Seo et al. [30], with some small modifications. Briefly, *E. coli* cells were treated with different amounts of TQ (from 0 and 512 µg/mL) and incubated for 3 h at 37 °C. Then, the cells were additionally incubated with DCFH–DA (500 μM in phosphate-buffered saline (PBS)) in a final concentration of 10 μM for 1 h at room temperature in the dark. The amounts of ROS were measured by fluorescence spectrophotometry (JASCO FP-8500, JASCO, Tokyo, Japan); λex = 495 nm, λem = 525 nm).

### 4.7. Statistical Analyses

All experiments were performed in triplicates. Differences between means were analyzed with the Student’s *t*-test for independent samples with OriginPro 2022 and considered significant if *p* value was below 0.05.

## 5. Conclusions

In the course of presented work the protective effect of glutathione (GSH) on *Escherichia coli* cells grown in a high concentration of thymoquinone (TQ) was demonstrated. It was shown that GSH may play a very important role in the detoxification of thymoquinone. Its presence and ease of conjugation with TQ and its derivatives increase the lethal concentration of TQ for a cell. It also suggests that GSH may play a similar role in the presence of other quinones and their derivatives.

## Data Availability

Not applicable.

## References

[1] Loi V.V., Rossius M., Antelmann H. (2015). Redox regulation by reversible protein S-thiolation in bacteria. Front. Microbiol..

[2] Sies H. (1999). Glutathione and its role in cellular functions. Free Radic. Biol. Med..

[3] Griffith O.W. (1999). Biologic and pharmacologic regulation of mammalian glutathione synthesis. Free Radic. Biol. Med..

[4] Khalife K.H., Lupidi G. (2007). Nonenzymatic reduction of thymoquinone in physiological conditions. Free Radic. Res..

[5] Smirnova G.V., Muzyka N.G., Oktyabrsky O.N. (2005). Effects of cystine and hydrogen peroxide on glutathione status and expression of antioxidant genes in *Escherichia coli*. Biochemistry.

[6] Smirnova G., Muzyka N., Oktyabrsky O. (2012). Transmembrane glutathione cycling in growing *Escherichia coli* cells. Microbiol. Res..

[7] Smirnova G.V., Tyulenev A.V., Muzyka N.G., Oktyabrsky O.N. (2020). Study of the relationship between extracellular superoxide and glutathione production in batch cultures of *Escherichia coli*. Res. Microbiol..

[8] Helbig K., Bleuel C., Krauss G.J., Nies D.H. (2008). Glutathione and transition-metal homeostasis in *Escherichia coli*. J. Bacteriol..

[9] Gawron G., Krzyczkowski W., Łyżeń R., Kadziński L., Banecki B. (2021). Influence of Supercritical Carbon Dioxide Extraction Conditions on Extraction Yield and Composition of *Nigella sativa* L. Seed Oil-Modelling, Optimization and Extraction Kinetics regarding Fatty Acid and Thymoquinone Content. Molecules.

[10] Hannan M.A., Rahman M.A., Sohag A.A.M., Uddin M.J., Dash R., Sikder M.H., Rahman M.S., Timalsina B., Munni Y.A., Sarker P.P. (2021). Black Cumin (*Nigella sativa* L.): A Comprehensive Review on Phytochemistry, Health Benefits, molecular pharmacology, and safety. Nutrients.

[11] Malik S., Singh A., Negi P., Kapoor V.K. (2021). Thymoquinone: A small molecule from nature with high therapeutic potential. Drug Discov. Today.

[12] O’Brien P.J. (1991). Molecular mechanisms of quinone cytotoxicity. Chem. Biol. Interact..

[13] Kruk I., Michalska T., Lichszteld K., Kladna A., Aboul-Enein H.Y. (2000). The effect of thymol and its derivatives on reactions generating reactive oxygen species. Chemosphere.

[14] Bolton J.L., Trush M.A., Penning T.M., Dryhurst G., Monks T.J. (2000). Role of quinones in toxicology. Chem. Res. Toxicol..

[15] Armutcu F., Akyol S., Akyol O. (2018). The interaction of glutathione and thymoquinone and their antioxidant properties. Electron. J. Gen. Med..

[16] Hariharan P., Paul-Satyaseela M., Gnanamani A. (2016). In vitro profiling of antimethicillin-resistant Staphylococcus aureus activity of thymoquinone against selected type and clinical strains. Lett. Appl. Microbiol..

[17] Goel S. (2018). Mishra Thymoquinone inhibits biofilm formation and has selective antibacterial activity due to ROS generation. Appl. Microbiol. Biotechnol..

[18] Salmani J.M., Asghar S., Lv H., Zhou J. (2014). Aqueous solubility and degradation kinetics of the phytochemical anticancer thymoquinone; probing the effects of solvents. pH and light. Molecules.

[19] El-Najjar N., Chatila M., Moukadem H., Vuorela H., Ocker M., Gandesiri M., Schneider-Stock R., Gali-Muhtasib H. (2010). Reactive oxygen species mediate thymoquinone-induced apoptosis and activate ERK and JNK signaling. Apoptosis.

[20] Zubair H., Khan H.Y., Sohail A., Azim S., Ullah M.F., Ahmad A., Sarkar F.H., Hadi S.M. (2013). Redox cycling of endogenous copper by thymoquinone leads to ROS-mediated DNA breakage and consequent cell death: Putative anticancer mechanism of antioxidants. Cell Death Dis..

[21] Newton G.L., Fahey R.C., Rawat M. (2012). Detoxification of toxins by bacillithiol in *Staphylococcus aureus*. Microbiology.

[22] Di Monte D., Ross D., Bellomo G., Eklow L., Orrenius S. (1984). Alterations in intracellular thiol homeostasis during the metabolism of menadione by isolated rat hepatocytes. Arch. Biochem. Biophys..

[23] Goswami M., Mangoli S.H., Jawali N. (2006). Involvement of reactive oxygen species in the action of ciprofloxacin against *Escherichia coli*. Antimicrob. Agents Chemother..

[24] Badary O.A., Taha R.A., Gamal El-Din A.M., Abdel-Wahab M.H. (2013). Thymoquinone is a potent superoxide anion scavenger. Drug Chem. Toxicol..

[25] Di Monte D., Bellomo G., Thor H., Nicotera P., Orrenius S. (1984). Menadione-induced cytotoxicity is associated with protein thiol oxidation and alteration in intracellular Ca^2+^ homeostasis. Arch. Biochem. Biophys..

[26] Ahmad Z., Laughlin T.F., Kady I.O. (2015). Thymoquinone Inhibits *Escherichia coli* ATP Synthase and Cell Growth. PLoS ONE.

[27] Suzuki H., Koyanagi T., Izuka S., Onishi A., Kumagai H. (2005). The yliA, -B, -C, and -D genes of *Escherichia coli* K-12 encode a novel glutathione importer with an ATP-binding cassette. J. Bacteriol..

[28] Gawron G., Krzyczkowski W., Lemke K., Oldak A., Kadzinski L., Banecki B. (2019). Nigella sativa seed extract applicability in preparations against methicillin-resistant *Staphylococcus aureus* and effects on human dermal fibroblasts viability. J. Ethnopharmacol..

[29] Reed P., Atilano M.L., Alves R., Hoiczyk E., Sher X., Reichmann N.T., Pereira P.M., Roemer T., Filipe S.R., Pereira-Leal J.B. (2015). *Staphylococcus aureus* Survives with a Minimal Peptidoglycan Synthesis Machine but Sacrifices Virulence and Antibiotic Resistance. PLoS Pathog..

[30] Seo Y., Park K., Hong Y., Lee E.S., Kim S.S., Jung Y.T., Park H., Kwon C., Cho Y.S., Huh Y.D. (2020). Reactive-oxygen-species-mediated mechanism for photoinduced antibacterial and antiviral activities of Ag_3_PO_4_. J. Anal. Sci. Technol..

